# First Identification of *Trichinella pseudospiralis* in a Golden Jackal (*Canis aureus*) in Romania

**DOI:** 10.3390/pathogens13010032

**Published:** 2023-12-29

**Authors:** Ana-Maria Marin, Dan-Cornel Popovici, Gianluca Marucci, Simona Cherchi, Narcisa Mederle

**Affiliations:** 1 Faculty of Veterinary Medicine, University of Life Sciences “King Michael I”, 300645 Timisoara, Romania; anamaria.marin@usvt.ro (A.-M.M.); narcisamederle@usvt.ro (N.M.); 2 Forestry Faculty, Transilvania University Brasov, 500123 Brasov, Romania; 3 Department of Infectious Diseases, Istituto Superiore di Sanità, 00161 Rome, Italy; gianluca.marucci@iss.it (G.M.); simona.cherchi@iss.it (S.C.)

**Keywords:** trichinellosis, parasitic disease, zoonosis

## Abstract

*Trichinella* spp. are etiological zoonotic agents that spread throughout the world and affect mammals, birds, and reptiles. Within this genus, *Trichinella pseudospiralis* is the only recognized non-encapsulated species known to infect mammals and birds. This species has been reported in the majority of European countries, and the real epidemiological scenario of this species remains to be defined because its detection in mammals is much lower than that of the capsulated species. The aim of this study was to examine the presence of *Trichinella* larvae isolated from the muscles of a jackal from the hunting fund of 36 Murfatlar, Constanta County, Romania. The muscle samples were examined by artificial digestion, and the larvae were identified at the species level by multiplex PCR. The presence of larvae belonging to *T. pseudospiralis*, a species more frequently reported in carnivorous birds, was observed. This study describes the first identification of *T. pseudospiralis* in a jackal. The results suggest that there is an urgent need to investigate which species of mammals and/or birds act as reservoirs for this zoonotic nematode in Romania.

## 1. Introduction

The species of the genus *Trichinella* represents a group of nematodes distributed globally that affect an impressive number of hosts among mammals, birds, reptiles and humans [1]. Currently, 13 taxa are described in the *Trichinella* genus, named as the encapsulated species *T. spiralis*, *T. nativa*, *T. britovi*, *T. murrelli*, *T. nelsoni*, *T. patagoniensis*, *T. chanchalensis*, and *Trichinella* genotypes T6, T8 and T9, exclusively in mammals. Non-encapsulated species are *T. pseudospiralis*, which can infect mammals and birds, and *T. papuae* and *T. zimbabwensis*, which can infect mammals and reptiles [2]. In Europe, four species are present: *T. spiralis*, *T. nativa*, *T. britovi* and *T. pseudospiralis* [3,4]. In Romania, only two of these species have been detected to date, *T. spiralis* and *T. britovi*, the latter affecting especially wild animals [5,6,7,8].

The zoonotic risk species, *T. pseudospiralis*, is the only species that equally affects mammals and birds [9]. From its first report in a raccoon (*Procyon lotor*) specimen from Russia [10], *T. pseudospiralis* has been detected in domestic and wild fauna, in 18 species of mammals, including humans, and in eight species of birds, for a total of 249 isolates collected from America, Asia, Australia, and Europe [9]. In the European Union, *T. pseudospiralis* has been reported in 19 countries out of 28 and has been isolated from the muscles of ten mammalian hosts [9,11,12]. In Romania, *T. pseudospiralis* was isolated from the muscle of a domestic pig, according to data reported in the International *Trichinella* Reference Center database [12]. The risk for human health represented by this species is documented by the 128 cases of human infection [9] reported in New Zealand, Thailand, France, and Italy [13,14,15,16]. The species responsible for the infections was confirmed by molecular identification conducted by Ranque, S. et al. and by Gómez Morales, M.A. et al. [16,17]. The wide distribution across the globe and the impressive number of hosts (mammals and birds) compensate to some extent for the low prevalence that could limit the epidemiological value of zoonotic nematode infection [11].

In recent years, the golden jackal (*Canis aureus* L.) has become an invasive species with an increasingly obvious presence in Romania’s fauna. The data provided by the Ministry of the Environment, Waters and Forests regarding the size of the golden jackal population, and also its population dynamics [18] is eloquent. According to the annual evaluation studies of the herds of these animals hunting fauna in the last decade, the estimated herd has increased more than 3.8 times, from 7566 specimens in 2014 to 28871 specimens in 2023. This dynamic demonstrates once again, both the invasive and expansionist characteristics of the species, as well as the high degree of adaptability and ecological plasticity that this species registers in Romania and also in Europe [19,20]. A mesocarnivore, the golden jackal is present in large numbers in ecosystems where top predators (wolves and lynxes) are not present, and also where these large carnivore species are still present, playing an active role as a species providing ecosystem services [21]. The conditions favorable to the expansion of the golden jackal within this ecological niche include climate change, the transformation of natural habitats into agricultural habitat, and the hunting management measures taken against top predators, especially wolves [22]. Within this ecological niche, where predators at the top of the food pyramid are poorly represented or absent, the jackal has become a very important species that provides ecosystem services within this food chain [21,23].

Extensive territorial mobility, wide foraging opportunities, a generous geographical area, and a lack of natural predators make this mesocarnivore an important host for a variety of parasites, but also a natural sylvatic reservoir for *Trichinella* spp. [24,25,26]. The purpose of this study was to report the first molecular identification of the species *T. pseudospiralis* in jackals from Romania.

## 2. Materials and Methods

### 2.1. The Target Hosts

The golden jackal, a wild carnivore belonging to the Canidae family, has golden-yellow to reddish fur, a short tail, and large, pointed ears. This species is widespread in northern Africa, southern Asia, and southern Europe, including Romania. The food eaten by this mesocarnivore varies from plant species, mushrooms, and seasonal fruits to a variety of insect, amphibian, bird, and mammal species, and is directly influenced by the seasonal supply of the habitats it occupies [21,27]. This wild mammal has a defining role in the occupied habitat and provides numerous ecosystem services, such as facultative necrophagy and feeding on agricultural crop pests, especially rodents. This carnivore represents a host of some parasites with zoonotic potential, such as nematodes of the genus *Trichinella*.

### 2.2. Diagnostic Procedures

On 12 September 2023, three male jackals were shot based on the annual harvest quota approved by the Minister of the Environment, Water and Forests, number 1630/2023. The hunting action through which these jackal specimens were harvested was carried out in compliance with Law 407/2006 (on hunting and wildlife protection) [28]. These animals were harvested by hunters on the number 36 Murflatar hunting grounds, Constanța County, and transported under legal conditions to the Faculty of Veterinary Medicine/ University of Life Sciences in Timisoara.

About 30 g of muscle from the diaphragm and foreleg muscles were sampled from each animal and tested for the presence of *Trichinella* spp. larvae by the artificial digestion method according to the Commission Regulation (EC) no. 1375/2015 [29]. *Trichinella* spp. larvae collected from the digestive fluids were kept in 90 % ethyl alcohol and sent to the European Union Reference Laboratory for Parasites of the Istituto Superiore di Sanitá (ISS) (Rome, Italy) for species identification. DNA was extracted from single larvae using the DNA IQ System Kit (Promega, Medison, WI, USA) and Tissue and Hair Extraction Kit (Promega, USA). Five primer sets, targeting specific regions (Expansion Segment V, ITS1 and ITS2) of the ribosomal DNA repeats, were used in a multiplex PCR to obtain a species–specific electrophoretic DNA banding pattern [30,31]. PCR products were purified using the QIAquick PCR Purification Kit (Qiagen, Hilden, Germany) according to the manufacturer’s instructions and sent to Eurofins Scientific (Luxembourg, Luxembourg) Company for standard Sanger sequencing. The Basic Local Alignment Search Tool (BLAST) was used to compare the ESV sequence to the GenBank database for species confirmation. The bioinformatics platform CLC Workbench 8.0.1 (Qiagen, Hilden, Germany) was used to align the ESV sequence versus its homologous sequence obtained from *T. pseudospiralis* isolates belonging to Palearctic, Nearctic, and Australian populations. A total of seven Palearctic isolates (Bulgaria, Denmark, Finland, Italy, Kamchatka, Romania, and Southern Russia), one Nearctic isolate (Alabama), and one Australian isolate (Tasmania) were used in the alignment (Table 1).

## 3. Results

One out of the three *C. aureus* specimens collected from Constanța County (44°16′75″ N; 28°38′09″ E) tested positive for the presence of *Trichinella* spp. larvae. Larvae were identified as *T. pseudospiralis* by multiplex PCR. The amplified ESV sequence (OR916274) showed a 100% identity with the homologous sequence obtained from *T. pseudospiralis* larvae collected in the Caucasus region (S82661.1).

**Figure 1:**
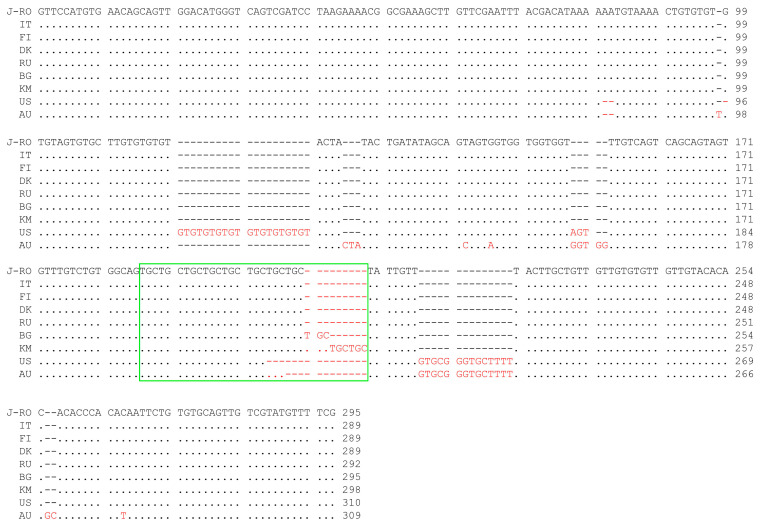
Alignment of homologous ESV sequences of *T. pseudospiralis* isolate belonging to Palearctic, Nearctic, and Australian populations. J-RO, isolate from the jackal of Romania (ISS9492); IT, isolate from a wild boar hunted in Northern Italy (ISS2851); FI, isolate from a raccoon dog (*N. procyonoides*) from Finland (ISS681); DK, isolate from an American mink (*N. vison*) from Denmark (ISS1938); RU, isolate from a raccoon dog (*N. procyonoides*) from Southern Russia (ISS13); BG, isolate from a red fox (*V. vulpes*) from Bulgaria (ISS1707); KM, isolate from a brown rat (*R. norvegicus*) from Kamchatka (ISS588); US, isolate from a black vulture (*C. atratus*) from the USA (ISS470); AU, isolate from a tiger cat (*D. maculatus*) from Australia (ISS141). Conserved bases are represented by dots; gaps are represented by dashes; different residues are highlighted in red; the TGC microsatellite region is boxed in green.

The ESV sequence of *T. pseudospiralis* collected from the golden jackal was shown to be identical to that obtained from some Palearctic isolates (collected in Denmark, Finland, and Italy) and to differ from that of other Palearctic isolates (collected in Bulgaria and Russia) only for the number of repeats of the microsatellite TGC (Figure 1). TGC microsatellite length polymorphism represents a peculiar characteristic of the Palearctic isolates of *T. pseudospiralis* [32]. The high variability of the microsatellite region can explain the differences observed between the ESV sequences in the different European isolates.

## 4. Discussion

The golden jackal is predominantly distributed in south-eastern Europe and has expanded its range to include parts of Italy, France, Switzerland, Germany, and several other countries [22]. Larvae of *T. spiralis* [24,33] and *T. britovi* [33,34,35] were identified in the muscles of the jackals. In Romania, Blaga et al. isolated, for the first time, *T. britovi* larvae from jackal muscle in 2008 [36]. A study by Pozio, E. et al. in 2009 helped to better define the hosts and habitats of *T. britovi* and *T. spiralis* in Europe, but limited information is available regarding *T. pseudospiralis* [4]. According to what has been so far reported in the literature, this study reveals the first identification of *T. pseudospiralis* in a jackal. Infections from this species have been reported in hosts such as birds and wild mammals. The first identification of *T. pseudospiralis* in central Europe was made in East Slovakia, in 2005, in pigs, rats, and cats [37]. Later, the parasite was observed in birds of prey; in 2021 the presence of *T. pseudospiralis* was reported in wild boars in Slovakia [38] and Estonia [39].

In Italy, *T. pseudospiralis* was reported in the following species: wild boar (*S. scroafa*) [40], wolf (*Canis lupus italicus*) [41], red kite (*Milvus milvus*) [42] and western marsh harrier (*Circus aeruginosus*) [43].

The first identifications of *T. pseudospiralis* in red foxes (*V. vulpes*) were reported in Germany [44], Poland [45] and UK [46]. In Central Europe, Cybulska A. et al., identified *T. pseudospiralis* larvae in raccoons (*P. lotor*) for the first time in 2018 [47]. *T. pseudospiralis* was isolated from the muscles of domestic pigs originating from Croatia [48], and Bosnia and Herzegovina [49]. In wild animals in Scandinavian regions, *T. nativa*, *T. britovi*, *T. spiralis* and *T. pseudospiralis* were identified, the latter being isolated from the muscles of wild boars and lynxes [50]. In Germany, in 2006, the first *T. pseudospiralis* and *T. spiralis* mixed infection was observed in wild boar muscle tissue [51] and, later, *T. pseudospiralis* was identified in wild boars in Hungary [52], the Iberian Peninsula [53], and Croatia [54].

On the American continent, *T. pseudospiralis* larvae were isolated from a wild boar [55], and jaguars and lynxes are considered reservoir hosts of this species [56,57]. In northern Canada, in 2019 [58], *T. pseudospiralis* was identified in a wolverine (*Gulo gulo*). In the neotropical region, after the identification of the encapsulated species, *T. spiralis* and *T. patagoniensis*, the presence of *T. pseudospiralis* was also reported in a domestic pig [59].

Until now, *T. pseudospiralis* had been detected in Romania only in the muscles of domestic pigs originating from the counties of Constanța (in 2016), and Mureș (in 2017) [12]. Nevertheless, a wide range of hosts can be affected by this non-encapsulated species, and very few cases of infection are reported compared to those involving the encapsulated *Trichinella* species. It is noteworthy that the jackal infected with *T. pseudospiralis*, the object of this study, was hunted in the same county (Constanta) from which in 2016, the same *Trichinella* species was detected in a domestic pig for the first time in Romania. We can speculate about the presence of a *T. pseudospiralis* population circulating in the wild fauna of Constanța County that may be a potential source of infection for domestic animals and consequently represents a potential risk for human health. Humans can acquire *Trichinella* infections from eating raw meat, foods such as pork, or wild game (e.g., badger, bear, and wild boar) [60]. In Romania, cases of human trichinellosis have been reported, both in children and in adults; to date, the only species involved in the etiology was *T. spiralis* [61,62].

The increasing the number of wild boars and red foxes, the spread of the raccoon dog from eastern to western Europe, and of the jackal from the south-east to the north-west of Europe contributes to the increase in the prevalence of *Trichinella* circulating in wildlife [25,63]. In recent decades, areas with golden jackals have increased significantly in Europe after population extinction in the first half of the 20th century. Currently, there is an acceleration in the rate of population growth in Bulgaria and Greece and the emergence of new populations in Turkey, Ukraine, Romania, Serbia, Croatia, Slovenia, Hungary, and Austria [64]. In Italy, Slovakia, Germany, and the Czech Republic, vagrant individuals are registered [64]. Moreover, jackals migrate for long distances through natural ecological corridors and thus become factors involved in the long-distance spread of zoonotic parasites in non-endemic areas of Europe [25,26,64]. In this context, another possible explanation for the presence of the *T. pseudospiralis* larvae in the jackal object of this study is that the animal could have acquired the infection in Bulgaria, where this parasite has been reported in specimens of wild boar, badgers, and foxes.

The jackal’s role as a wild reservoir for human parasites is supported in Romania by Gherman, C. et al. Jackals interact in their habitat not only with other wild carnivores but also with domestic animals, with which they share a variety of parasitic species. This phenomenon may be associated in the future with the territorial expansion of various parasitic diseases [24].

## 5. Conclusions

According to the author’s knowledge, this study reports on the first case of *T. pseudospiralis* infection in the golden jackal. Further studies will be necessary to define the role of this host in the maintenance and spread of *T. pseudospiralis* in wild fauna and its role as a source of infection for domestic animals. Based on the biological characteristics and behavioral traits of the golden jackal and its demographic and territorial expansion, we could expect, in Romania and in Europe, a change in the dynamics of this parasitic species, especially the zoonotic ones.

## Figures and Tables

**Table 1:** *Trichinella pseudospiralis* isolates from Australian, Nearctic, and Palearctic regions.

Isolate Code ^a^/An ^b^	Original Host	Geographical Origin
ISS13/S82661.1	Raccoon (*P. lotor*)	Caucasus region (Russia)
ISS141	Tiger cat (*Dasyurus maculatus*)	Tasmania (Australia)
ISS470/S82657.1	Black vulture (*Coragypus atratus*)	Alabama (USA)
ISS588	Brown rat (*Rattus norvegicus*)	Kamchatka (Russia)
ISS681	Raccoon dog (*Nyctereutes procyonoides*)	Finland
ISS1707	Red fox (*Vulpes vulpes*)	Bulgaria
ISS1938	American mink (*Neogale vison*)	Denmark
ISS2851	Wild boar (*Sus scrofa*)	Italy
ISS9492/OR916274	Golden jackal (*C. aureus*)	Romania

^a^ Isolate code of the International Trichinella Reference Centre, Rome, Italy; ^b^ GenBank accession number.

## Data Availability

All data related to this study are presented and published here.
